# Systematic review and meta‐analysis of antipsychotic discontinuation in dementia

**DOI:** 10.1002/trc2.70188

**Published:** 2025-12-15

**Authors:** Sophie Roche, Nimesh Naran, Janneke Scholtz, Kathy Y. Liu, Suzanne Reeves, Rob Howard

**Affiliations:** ^1^ North London NHS Foundation Trust St Pancras Hospital London England; ^2^ Division of Psychiatry University College London London England

**Keywords:** Alzheimer, antipsychotic, dementia, discontinuation, meta‐analysis

## Abstract

**INTRODUCTION:**

Antipsychotics are used to treat behavioral and psychological symptoms of dementia (BPSD). However, treatment is associated with adverse outcomes. A 2018 Cochrane review found low‐quality evidence that discontinuation may have little effect on BPSD by primarily comparing the difference in number of study non‐completers between treatment groups and did not report a pooled effect size for relapse risk. We performed a meta‐analysis of the pooled risk ratio (RR) of relapse of symptoms following antipsychotic medication discontinuation in people with dementia. We hypothesized that trial design (enriched responder samples vs long‐term user withdrawal), treatment duration, length of follow‐up, withdrawal speed, and sex of participants would affect relapse risk.

**METHODS:**

We systematically searched PsycINFO, EMBASE, PubMed, and ClinicalTrials.gov databases for studies published between January 2018 and June 2024. The eight papers identified in the 2018 Cochrane review were also included in the meta‐analysis. The study was registered with PROSPERO (CRD42024570329).

**RESULTS:**

A total of nine studies were included, providing 682 observations with 135 events. Relapse definitions included participant removal due to worsening behavior or starting regular prescription of antipsychotic/rescue medication. A random‐effects model found a pooled RR of relapse of 1.52 (95% CI: 1.18 to 1.95, *p* = 0.005) with stopping antipsychotics. Meta‐regressions showed no effect of trial type, withdrawal speed, length of follow‐up, or participant sex on RR.

**DISCUSSION:**

While it is important to prescribe cautiously and to reduce and withdraw prescriptions in those who are considered to no longer need antipsychotics, there is a subgroup of patients for whom continuing medication is important. It was particularly surprising that the relative risk of relapse was unaffected by trial design, with similar effect sizes in both trials aiming to reduce prescription and prove efficacy. Further research is needed to identify factors associated with successful antipsychotic withdrawal.

**Highlights:**

- An updated meta‐analysis of trials of withdrawal of antipsychotics in dementia.
- Relative risk of symptom relapse increased when stopping antipsychotics.
- Relapse risk was not affected by trial design or prior length of treatment.
- Relapse risk was not affected by length of follow‐up, withdrawal speed, or sex.

## 1 BACKGROUND

The World Health Organization estimated that in 2023, dementia affected more than 55 million people worldwide, with over 10 million new cases every year.^1^ In the UK, the prevalence rate of dementia in older people is estimated to be 7.1%,^2^ and, with an aging population, the number of those at risk is increasing.^2^, ^3^ In addition to cognitive impairment, dementia can present with so‐called behavioral and psychological symptoms of dementia (BPSD), and these can include psychosis (delusions and hallucinations), agitation, emotional lability, depression, anxiety, and disinhibition.^4^ More than 90% of people with dementia will experience BPSD at some point over the life course of their illness.^5^

BPSD presents a challenge in terms of clinical management. Although non‐pharmacological and psychosocial management should be fully explored as first line and alongside any eventual medications,^6^, ^7^ the options for pharmacological treatment when required are limited. Current National Institute for Health and Care Excellence (NICE) Guidance only supports antipsychotic drug prescribing in people with dementia when they are “at risk of harming themselves or others or are experiencing agitation, delusions or hallucinations that are causing them severe distress.”^8^ Risperidone and haloperidol are the only antipsychotics licensed in the UK for treating non‐cognitive symptoms of dementia.^7^ Internationally, brexpiprazole was approved by the United States Food and Drug Administration in 2023 for treatment of agitation associated with dementia due to Alzheimer's disease.^9^ NICE Guidance advises treatment with antipsychotics with the lowest effective dose for the shortest possible time, with reassessment after 6 weeks to check whether medication is still required^10^ and 3‐monthly follow‐up thereafter. Antipsychotic use in dementia is increasingly associated with adverse events^11^ and increased mortality.^12^, ^13^ Cautious prescribing is therefore advised, with an aim to reduce prescription in those who are considered to no longer need treatment. However, questions remain about how to identify when this is the case, what optimal withdrawal strategies might be, and how best to weigh the risk of relapse against the risk of adverse physical health outcomes with continued treatment.

A 2018 Cochrane review aimed to evaluate whether withdrawal of antipsychotic agents had been successful, to list the different strategies employed for withdrawal, to measure the effects of withdrawal on participants' behavior, and to assess safety.^14^ The review found low‐quality evidence that discontinuation may have little effect on BPSD by primarily comparing the difference in number of study non‐completers between groups and did not report a pooled effect size for relapse risk.

We performed a meta‐analysis of the pooled relative risk (also known as relative risk) of relapse when antipsychotic medications are discontinued in people with dementia. We were particularly interested in whether trial design influenced risk of relapse. Antipsychotic withdrawal trials have broadly been conducted in two ways. The first involves a “randomized withdrawal in responders” design, where a new antipsychotic agent is given for a set period, and treatment responders are then randomized to continue or withdraw from the drug. The second involves a “randomized cessation in chronic treatment” design, where asymptomatic patients on long‐term antipsychotic treatment are randomized to continue or withdraw. We hypothesized that the different nature of participant groups in these trials, with the first studying an enriched responder group, early in the course of antipsychotic treatment, would result in a significant increase in the risk ratio (RR) of relapse. We also hypothesized that treatment duration, length of follow‐up, withdrawal speed, and participant sex would affect relapse risk.

## 2 METHODS

The methodology for this review was structured in accordance with Preferred Reporting Items for Systematic Reviews and Meta‐Analyses (PRISMA) guidelines.^15^ The protocol was registered in the PROSPERO database (CRD42024570329). We systematically searched PsycINFO, EMBASE, PubMed, and ClinicalTrials.gov from January 2018 to June 2024. We validated our search terms by ensuring they included the eight papers identified in the Cochrane 2018 review. The final search strategy terms were as follows: (dementia OR “nursing home”)|Other terms: (antipsychotic* OR anti‐psychotic* OR neuroleptic* OR phenothiazine* OR butyrophenone* OR risperidone OR Risperdal OR olanzapine OR haloperidol OR prothipendyl OR methotrimeprazine OR clopenthixol OR flupenthixol OR clothiapine OR metylperon* OR melperon* OR droperidol OR pipamperone OR dipiperon OR benperidol OR anquil OR bromperidol OR bromidol OR flusperilene OR pimozide OR orap OR penfluridol OR sulpiride OR veralipride OR levosulpiride OR sultopride OR aripiprazole OR clozapine OR quetiapine OR thioridazine) AND (cessation OR withdrawal OR discontinu* OR taper* OR stop*) AND (trial OR double‐blind OR randomi*)|First posted on or after January 1, 2018.

Two authors (Sophie Roche and Janneke Scholtz) independently screened the titles and abstracts using the following inclusion criteria: (1) quantitative research, reporting relapse RR (or numbers who relapsed in each group), (2) diagnosis of dementia or study of nursing home residents, (3) withdrawal or discontinuation of antipsychotics for treatment of BPSD (including psychosis and agitation), and (4) randomized controlled trials.

Studies were excluded if (1) antipsychotic initially started for alternative functional psychiatric diagnosis (e.g., schizophrenia) or (2) open‐label treatment throughout trial. Decisions were discussed and conflicts resolved between reviewers in consensus meetings. Full‐text articles of the selected abstracts were then reviewed closely, again independently by Sophie Roche and Janneke Scholtz. A final decision meeting was then held to decide ultimate article inclusion.

Two authors (Sophie Roche and Nimesh Naran) independently extracted data from included studies. The following data were extracted:

1. First author, publication year, and journal.
2. Number, age, and sex distribution of participants.
3. Distribution of dementia subtype.
4. Duration of discontinuation period and open‐label treatment period if applicable.
5. Withdrawal speed (abrupt vs tapered withdrawal).
6. Trial design (whether antipsychotics started then stopped in context of this trial).
7. Baseline severity of neuropsychological symptoms (NPS).
8. Definition of relapse.
9. Number of relapses in each group.
10. Number of dropouts in each group.
11. Requirement of rescue medication.
12. Presence of withdrawal symptoms.
13. Primary and secondary outcomes as defined by paper.

**RESEARCH IN CONTEXT**

1. **Systematic review**: We systematically reviewed the literature using online databases and trial registries looking at withdrawal trials of antipsychotic medication in people with dementia.
2. **Interpretation**: Discontinuation of antipsychotic drugs in older people with dementia increased the relative risk of relapse of targeted treated symptoms. Relative risk of relapse was not affected by trial design, with equivalent effect sizes in both trials used to support reduction of prescription and demonstrate treatment efficacy. Relapse risk was not affected by treatment duration, length of follow‐up, withdrawal speed, or participant sex. Conclusions are limited by small study numbers and small numbers of participants in individual trials.
3. **Future directions**: Further studies are needed to identify those at higher risk of relapse following antipsychotic medication discontinuation and to improve precision around the increase in risk of relapse.

Where available, relapse was defined within the original articles. In the absence of any definition included in the original paper, relapse was defined as study withdrawals due to behavioral worsening or requirement of regular prescription of antipsychotic or rescue medication according to the data available in the article. Van Leewen et al.^14^ defined success of antipsychotic withdrawal as the ability to not drop out from the trial due to worsening of neuropsychiatric symptoms or no relapse to antipsychotic drug use during the trial. We widened this definition to additionally include the use of rescue medication such as clomethiazole and clonazepam, considering use of as required medication as a proxy for agitation.

Risk of bias was assessed for any newly selected studies using the Cochrane Risk of Bias 2 tool.^16^ Two authors (Sophie Roche and Nimesh Naran) independently assessed and discussed overall judgment. Papers included in the Cochrane 2018 review had risk of bias assessed by the authors at the time, and this was carried forward.

Meta‐analysis was performed using the “meta,” “dmetar” (version 0.1.0), “tidyvers,” and “readxl” (version 1.4.3) packages in Rstudio (version 2024.09.1 + 394).^17^, ^18^, ^19^, ^20^, ^21^, ^22^ Study control and intervention group numbers were extracted and relapse numbers for each group calculated. Pooled RR was calculated using random‐ and fixed‐effects models. Between‐study heterogeneity was assessed using Cochran's *I* ^2^.

The influence of trial type, trial length, withdrawal speed, and sex was assessed by meta‐regression.

Finally, a post hoc analysis was performed to investigate the absolute risk (AR) of relapse following antipsychotic withdrawal. The AR difference was calculated, then meta‐analysis and meta‐regression were performed as above.

## 3 RESULTS

**FIGURE 1:**
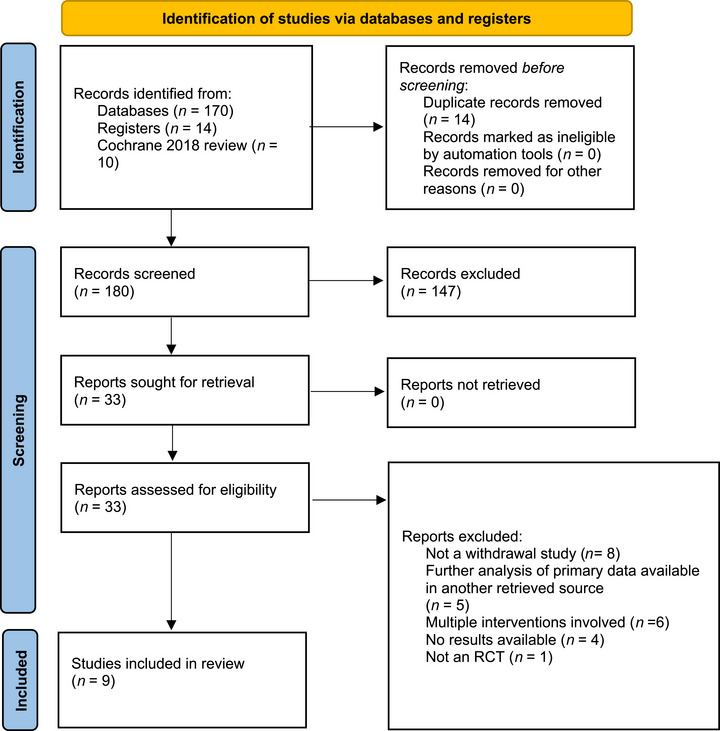
PRISMA flow diagram for the systematic review.

The initial search yielded 170 individual articles, of which 23 were retained after the initial screening of abstracts. Full‐text review of these articles led to 22 being discarded, primarily because they were not withdrawal studies or provided further analyses of data already available in another retrieved source. The selection process is shown in the flowchart in Figure 1.

When added to the eight articles identified in the 2018 Cochrane review, a total of nine articles were eligible to be included in analyses.^23^, ^24^, ^25^, ^26^, ^27^, ^28^, ^29^, ^30^, ^31^ One paper (Cohen‐Mansfield 1998) included in the 2018 review was not included in this analysis, as it did not distinguish those prescribed only lorazepam from those given antipsychotic medications. It also did not include information on relapse rates or study withdrawals due to behavioral worsening or requirement of regular prescription of antipsychotic/rescue medication. Another study (Bergh 2011) included in the 2018 Cochrane review was unpublished, and we were unable to access it for review.

A total of 682 patients were included, of whom 359 underwent antipsychotic withdrawal. A total of 124 patients across three studies were involved in a “randomized withdrawal” protocol. The remaining 235 patients across six studies followed a “cessation study” protocol. Withdrawal speed ranged from stopping immediately^23^, ^24^, ^29^, ^30^ to a maximum of a 2‐week tapering period. Length of follow‐up ranged from 4 weeks^29^ to 12 months.^23^ Gender distribution ranged from roughly equal (placebo group 47% male^31^) to an entirely female cohort.^28^ The remainder of the study characteristics are summarized in Table 1. Relapse was defined as recurrence of psychosis demonstrated by increases in baseline neuropsychiatric scores in three papers.^26^, ^27^, ^30^ Use of other antipsychotics for the treatment of dementia‐related psychosis and time to trial discontinuation were also included in Tariot's relapse definition. The remaining six papers did not give a specific definition of relapse. Characteristics related to outcomes and relapse are shown in Table 2.

**TABLE 1:** Summary of study characteristics from data extraction.

Author, year	Age, years	Sex (percentage of females)	Dementia subtype	Baseline severity of NPS	Duration of discontinuation period/open‐label treatment period	Trial design	Withdrawal speed	Number of participants
Ballard, 2004^24^	P: 83.6 (9.3), A: 83.1 (7.1)	P: 87%; A: 75.9%	Probable or possible Alzheimer's disease	NPI total score P: 16; A: 14	3 months	CS	Immediate	100
Ballard, 2008^23^	A: Average 84.8 (68.3–100.2)****; P: average 84.9 (67.0–100.6)	A: 77.1%; P: 75.6%	Possible or probable AD	Total NPI score; A: 17.4, P: 15.8	12 months discontinuation	CS	Immediate	A total of 165 randomized, 128 commenced treatment, 51 patients per arm analyzed for primary outcome.
Bridges‐Parlet, 1997^25^	P: 81.7 ± 6, A: 83.9 ± 6.1	P: 82%, A: 79%	Twenty‐three had diagnosis of AD (one of these also had diagnosis of PD and Korsakoffs), 13 just dementia	Physically aggressive behavior—W 3.9 incidents in 8 h, NW 2.2 incidents in 8 h.	4‐week withdrawal	CS	Five participants tapered during week 1 if dose equivalent > 50 mg of chlorpromazine	36
Devanand, 2011^26^	Mean age 75 (before responders identified and randomization)	57% (before responders identified and randomization)	Probable AD	BPRS 26.6 at start, 19.0 in responders by end of phase A	20 weeks open label, then 24 weeks discontinuation	RW	Two‐week tapering period	Twenty‐two responders from phase A continued to randomization
Devanand, 2012^27^	79.6 at baseline	Baseline 59%	Dementia, probably Alzheimer's disease	NPI 36.1 ± 17.0	16 to 32 weeks open label, 48 weeks total	RW	One‐week tapering if receiving 2 mg+ daily	A total of 112 responders in phase A, 110 randomized
Findlay, 1989^28^	>65 as inclusion criteria, does not mention distribution	100%	Senile dementia and Alzheimer type (ICD‐9).	Active SCAGS 79.6 LPRS 39.5; placebo 76.7, 34.6	4 weeks	CS	One week half dose	36
Ruths, 2008^29^	Mean age 84.1	A: 74%, P: 82%	Diagnosis of dementia according to ICD‐10, nursing home resident.	NPI mean score (SD) in intervention = 8.6 TAU = 7.9	4 week discontinuation	CS	Immediate	55
Tariot, 2021^30^	74.5 ± 8.3 (experimental) 73.8 + 08.4, placebo 74.9 ± 8.6)	58.4% in open phase (exp 59%, placebo 61.6%).	66.3% AD, 7.1% DLB, 1.8% FTD, 15.1% PD, 9.7% VD	SAPS‐H+D score 24.4 ± 9.2, CGI‐S score 4.7 ± 0.7	Twelve weeks pimavanserin open label, 26 weeks discontinuation.	RW	Immediate	A total of 392 open label, of whom 217 responded to pimavanserin (105 continued 112 switched to placebo)
Van Reekum, 2002^31^	Placebo 84.4 (4.6), active 82.9 (6.9)	P: 52.9, A: 43.7%	P: 10 AD, four vascular, three NOS; A: eight AD, five vascular, three NOS	NPI score P: 12.6 (11.3), A: 17.4 (12.5)	6 months	CS	Two‐week dose reduction period	34

Abbreviations: A, active treatment group; AD, Alzheimer's disease; BPRS, Brief Psychiatric Rating Scale; CGI‐S, clinical global impression–scale; CS, cessation study; DLB, dementia with Lewy bodies; EPS, extra pyramidal side effects; FTD, frontotemporal dementia; ICD, International Classification of Diseases; LPRS, London Psychogeriatric Rating Scale; NOS, not otherwise specified; NPI, Neuropsychiatric Inventory; NW, non withdrawal group; P, placebo group; PD, Parkinson's disease; RW, randomized withdrawal; SAPS H+D, Scale for the Assessment of Positive Symptoms—Hallucinations and Delusions; SCAGS, Sandoz Clinical Assessment‐Geriatric Scale; SD, standard deviation; TAU, treatment as usual; TD, tardive dyskinesia; VD, vascular dementia.

**TABLE 2:** Study characteristics related to outcomes and relapse.

Author, year	Definition of relapse	No. relapses	No. drop‐outs	Use of rescue medication	Withdrawal symptoms	Primary and secondary outcomes as defined by paper
Ballard, 2004^24^	Not defined	P: 6, A: 5	P: 14, A: 14	Withdrawn from study to receive rescue medication	Not quantified by participant – patients with baseline NPI scores at or below median (≤14) had particularly good outcome, with significantly greater reduction of agitation in patients receiving placebo, while patients with higher baseline NPI scores were significantly more likely to develop marked behavioral problems if discontinued from neuroleptics	Change in NPI total score, or key psychiatric/behavioral factors of agitation/mood/psychosis, remaining “stable” over treatment period
Ballard, 2008^23^	Not defined	Of those analyzed, three per group stopped allocated treatment due to behavioral deterioration	Thirteen lost to follow‐up in both groups, one of these was due to behavioral condition deterioration in active group but was not analyzed	Center coordinator decided whether patient needed to be withdrawn from study to receive additional “rescue” medication	Not specified	Primary: whether treatment with neuroleptic agents is associated with accelerated rate of cognitive decline in dementia. Secondary: (a) to examine impact of neuroleptics on function and other cognitive outcomes; (b) to determine whether discontinuing neuroleptics was associated with exacerbation of neuropsychiatric symptoms, both overall and in people with NPI scores above and below 14; (c) to examine impact of parkinsonism; and (d) to determine impact on global clinician‐related outcome
Bridges‐Parlet, 1997^25^	Does not define relapse, main outcomes are completion of study, physically aggressive behavior and certain other behaviors.	Two patients restarted on medication, only one went back on neuroleptic	P: 2, A: 0	One in withdrawal group required clonazepam	Increase in physically aggressive behavior in week 1 almost entirely driven by one patient who was remedicated at end of week 2	Main outcomes are completion of study, physically aggressive behavior, and certain other behaviors
Devanand, 2011^26^	50% worsening in target symptoms and on the CGI‐C.	40% continuation and 80% placebo	Zero after randomization	No concomitant psychotropic medications were permitted	None specified	Risk of relapse following discontinuation of haloperidol after initially responding
Devanand, 2012^27^	NPI score increase of 30%‐ or 5‐point increase plus score of 6 or 7 on CGI.	G1 15, G2+G3 once started on placebo 38 (pooled)	G1 5, G2 3, and G3 4 discontinued early	Lorazepam at dose of <1 mg/day was permitted if needed	No significant difference in adverse events between groups. AE included EPSE, confusion, agitation/aggression	(1) Time to relapse during weeks 0 to 16 and 17 to 32; (2) EPSE, TD, somatic symptoms, cognitive status, and physical function
Findlay, 1989^28^	Does not define relapse.	One per group receiving regular chlormethiazole	Zero (however notes small study patient numbers and short study time may have affected power to detect this)	Chlormethiazole two per group regular prescription (one per group received), two in placebo STAT dose, one in active STAT dose.	Not seen	(Not clearly defined) difference in cognitive function, behavior, or physical state
Ruths, 2008^29^	Not defined	Two in intervention group withdrawn due to behavioral deterioration	Four intervention, three TAU “completed the study prematurely”	Was allowed in the protocol to treat worsening BPSD, “sedative use remained stable”	The abrupt drug discontinuation may have contributed to some of the observed discontinuation effects	(1) Successful discontinuation of antipsychotic, (2) changes (baseline vs 4 weeks) in BPSD total scores, individual symptom scores, and factor scores, as well as proportion improved/worsened behavior
Tariot, 2021^30^	Recurrence of psychosis: increased of baseline of at least 30% in SAPS‐H+D total score and a CGI score of 6 or 7, hospitalization due to dementia related psychosis, use of other antipsychotics for the treatment of dementia related psychosis (2) time to trial discontinuation.	Twelve in active, 28 in placebo	Trial stopped early due to positive efficacy – A: 26 discontinued early, P: 46 discontinued early	Not specified	Mentioned in definition of psychosis but not expanded upon	(1) Time from randomization to relapse of psychosis, (2) time to trial discontinuation for any reason
Van Reekum, 2002^31^	Does not define relapse.	Withdrawal due to exacerbation of behavioral symptoms: P: 23.5%, A: 18.8%	P: 10, A: 6	No significant differences in rate of use of lorazepam, mean rate less than one tablet per subject per study visit	Not explicitly mentioned – placebo group may be at ∼26% greater risk of having exacerbation of behavioral symptoms upon discontinuation of long‐term antipsychotic use	Difference in proportions between subjects whose behavior worsened clinically in each group, analysis to identify possible predictors of relapse, difference in mean scores in cognition/function

Abbreviations: A, active treatment group; AD, Alzheimer's disease; AE, adverse event; BPSD, behavioral and psychological symptoms of dementia; CGI‐S, clinical global impression–severity; CS, cessation study; DLB, dementia with Lewy Bodies; EPSE, extra pyramidal side effects; FTD, frontotemporal dementia; P, placebo group; PD, Parkinson's disease; RW, randomized withdrawal; SAPS H+D, Scale for the Assessment of Positive Symptoms—Hallucinations and Delusions; SCAGS, Sandoz Clinical Assessment‐Geriatric Scale; STAT, medication to be given immediately; TAU, treatment as usual; TD, tardive dyskinesia; VD, vascular dementia.

### 3.1 Risk of bias

**FIGURE 2:**
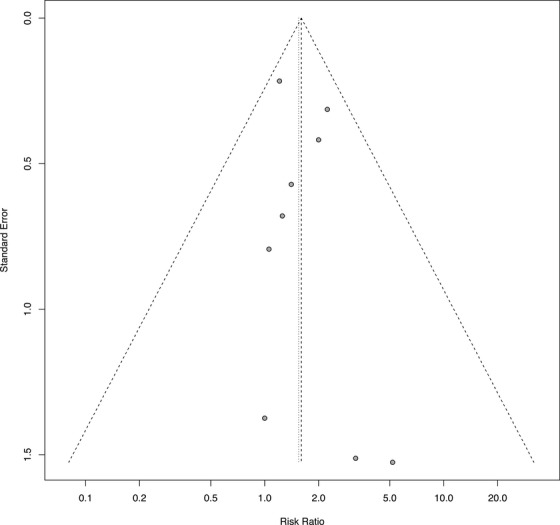
Funnel plot exploring publication bias.

The Rob2 tool for the newly included paper^30^ identified some concerns in domain 5 (selection of reported result) and domain 6 (overall bias). Publication bias was visually explored using funnel plots (Figure 2). Visual assessment of the funnel plot did not identify any concerns regarding publication bias.

### 3.2 Meta‐analysis

**FIGURE 3:**
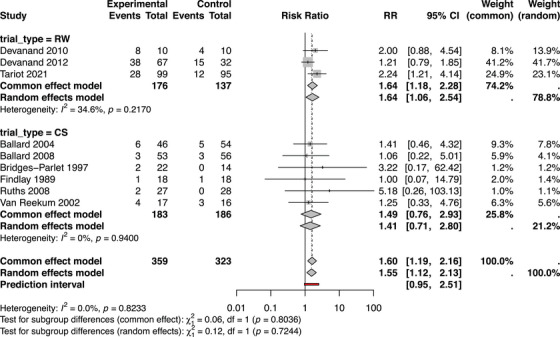
Forest plot of relative risk difference following antipsychotic withdrawal. CS, cessation study; Events, relapse events per group; RR, risk ratio; RW, randomized withdrawal trial; Total, sample size; Weight, adjusted fixed‐effects weight using Mantel–Haenszel method; 95% CI, associated 95% confidence interval.

Overall, the pooled studies demonstrated an increased relative risk of relapse in those patients who stopped antipsychotics, compared to those who continued (RR 1.54, 95% CI: 1.12 to 2.13, *p* = 0.008). The relative risk of relapse in individual studies and pooled is shown in Figure 3.

Meta‐regression found no effect of trial type, withdrawal speed, length of follow‐up, or participant sex on relative risk of relapse.

### 3.3 Post hoc analysis

After finding that there was no significant difference in relative risk when looking at trial type, we were interested in exploring, with post hoc analyses, whether there were any differences in absolute relapse risk related to trial type, trial length, withdrawal speed, or sex.

**FIGURE 4:**
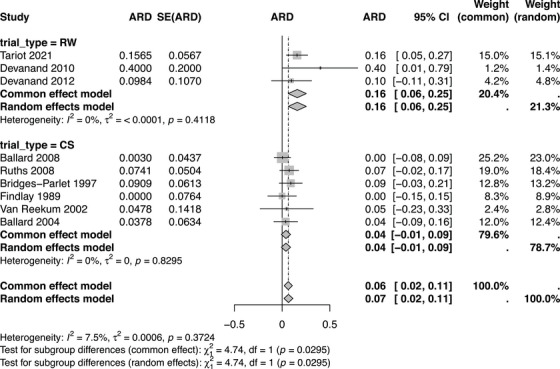
A forest plot of absolute risk (AR) difference following antipsychotic withdrawal. ARD, absolute risk difference; CS, cessation study; MD, mean difference; SE(MD), standard error of the mean difference; RW, randomized withdrawal trial; Weight, adjusted fixed‐effects weight using Mantel–Haenszel method; 95% CI, associated 95% confidence interval.

The mean AR of relapse associated with antipsychotic withdrawal was 32.8% compared to 19.8% in those who continued taking antipsychotic medication. Antipsychotic withdrawal resulted in relapse in 10.7% of those in cessation studies versus 54.9% in randomized withdrawal studies. For those who continued on their antipsychotic, the mean AR of relapse was 6.4% in cessation studies and 33.1% in randomized withdrawal studies. This is shown in Figure 4.

Meta‐regression showed no effect of withdrawal speed, length of follow‐up, or participant sex on AR of relapse.

## 4 DISCUSSION

We conducted a systematic review and meta‐analysis of relative risk of relapse after antipsychotic withdrawal in BPSD. We found that discontinuation of antipsychotics increased the overall relative risk of relapse by over 50%, regardless of trial design, participant sex, withdrawal speed, or length of follow‐up.

Surprisingly, we found no difference in the relative risk of relapse between the two alternative study methodologies that examined antipsychotic withdrawal in this participant population. It might be expected that those newly started on antipsychotic medication and selected on the basis of a positive response to treatment would be more likely to relapse following medication withdrawal. In randomized withdrawal studies, effect sizes in the withdrawal compared to continuation group have been used to argue for continuing treatment due to risk of relapse in some patients and has formed the basis of applications for marketing authorization.^30^, ^32^ Similarly, data from cessation studies have been used to justify reduction and withdrawal of treatment.^23^, ^24^, ^25^, ^28^, ^29^, ^31^ It is also worth considering that withdrawal studies are more likely to represent current practice, where antipsychotic prescribing is advised to be for as short an amount of time as possible before tapering and stopping.

Interestingly, post hoc analysis of the pooled data for AR of relapse did find a significant difference between study methodologies. We suggest that this is largely explained by a higher baseline relapse risk in the randomized withdrawal trials, as would be expected with their use of enhanced responder cohorts, compared to those on antipsychotic treatment for many years. This is an important distinction and argues for a more cautious approach to antipsychotic withdrawal in those who have only recently responded to drug treatment.

This analysis builds on work started in the 2018 Cochrane study, going further to pool data on relapse rates.^14^ We anticipated concerns that some studies might not have defined relapse of symptoms with our own definition, allowing us to source data from the text as appropriate. A strength of this method was that we were able to identify relapse rates across all of the included studies, regardless of differences in rating scales or medications used. However, there is a possibility that the studies were not powered to calculate relapse if this was not included as part of their original aims. It is also worth considering whether acute withdrawal or discontinuation states could be perceived as relapse, particularly in studies with a short or no tapering period and shorter follow‐up periods.

Other limitations include the small number of included studies with only three randomized withdrawal compared to six cessation studies, as well as the heterogeneity of study methods. Although we found no differences when looking at potential contributory factors, it is possible that the studies were not powered to detect these differences. The strengths of this study are the extensive and up‐to‐date database search, including trial registry data. The pooling of data allowed for the analysis of larger participant numbers than individual trials. The meta‐analysis found high homogeneity, demonstrating consistent effect size across studies, supporting the conclusions that we have drawn.

While it is important to prescribe cautiously and to reduce prescriptions when it is considered that antipsychotics are no longer needed, the finding of a 52% higher relative risk of relapse indicates a subgroup of patients for whom these medications are of ongoing importance to manage symptoms. Prescribers therefore should consider this when weighing the risks versus benefits of continuation for an individual patient, particularly considering how relapse risk may be lower for someone free of symptoms for a longer period of time compared to someone more recently stabilized. We also suggest that any decision to taper or stop an antipsychotic must be accompanied by appropriately robust monitoring for a return of symptoms that both are distressing and potentially put the patients and others at risk of harm. We were unable to identify factors of risk of relapse from the data available to us, and further research is required to identify factors that contribute to or reduce the chance of successful withdrawal. We have also demonstrated that current methodologies for testing effectiveness of antipsychotics, in terms of either randomized withdrawal studies or cessation studies, do not provide us with clear‐cut answers to support clinical decisions around stopping or continuing antipsychotic medications on an individual basis. Future research is required and may include large, high‐quality double‐blind withdrawal trials in those who have been on antipsychotics for a significant length of time and no longer experience non‐cognitive symptoms of dementia. Additional value would be added if these could be powered to investigate patient features associated with symptom relapse on medication withdrawal, as these could not be identified with currently available data.

## CONFLICT OF INTEREST STATEMENT

Sophie Roche is a contributor to intellectual property licensed by Oxford University Innovation to AstraZeneca. Suzanne Reeves, Rob Howard, and Kathy Y. Liu are supported by the University College London Hospitals’ National Institute for Health Research Biomedical Research Centre. Nimesh Naran and Janneke Scholtz have no conflicts of interest to declare. Author disclosures are available in the Supporting Information.

## CONSENT STATEMENT

No consent was required for the research reported herein.

## Supporting information



Supporting Information
